# Structural determinants of miR156a precursor processing in temperature-responsive flowering in Arabidopsis

**DOI:** 10.1093/jxb/erw248

**Published:** 2016-06-21

**Authors:** Wanhui Kim, Hee-Eun Kim, A Rim Jun, Myeong Gyo Jung, Suhyun Jin, Joon-Hwa Lee, Ji Hoon Ahn

**Affiliations:** ^1^Creative Research Initiatives, Department of Life Sciences, Korea University, Anam-dong 5-ga, Seongbuk-Gu, Seoul 136–701, Republic of Korea; ^2^Department of Chemistry and RINS, Gyeongsang National University, Jinju, Gyeongnam 660–701, Republic of Korea

**Keywords:** Ambient temperature, Arabidopsis, flowering time, miRNA processing, miR156, miR172, structural determinants.

## Abstract

MicroRNAs originate from primary transcripts (pri-miRNAs) containing hairpin structures. Plant pri-miRNAs have highly variable structures and little is known about the information encoded in their secondary structures. Arabidopsis miR156 is an ambient temperature-responsive miRNA and plays an important role in regulating flowering time. To identify the structural determinants for miR156 processing, we analyzed the effects of mutations introduced in the upper stem of pri-miR156a on its temperature-dependent processing and flowering time. The levels of pri-miR156a and mature miR156 were opposite at different temperatures. Mutations in the upper stem, especially the region closer to the miR156a/miR156a* duplex, reduced miR156 processing at 23 °C and 16 °C and caused a less severe phenotype compared with the un-mutated construct. Mutation in the second stem near the first cleavage site of pri-miR156a affected miR156 processing at 23 °C, but not at 16 °C. This was also seen in pri-miR172a, another ambient temperature-responsive miRNA. Replacement of the upper stem of pri-miR156a with that of pri-miR172a severely affected miR156 processing and flowering time. These results suggested that the upper stem of pri-miR156a is important for miR156 processing at different temperatures. In particular, the second stem adjacent to the first cleavage site plays a role in the regulation of ambient temperature-responsive flowering.

## Highlight

In Arabdiposis, the upper stem of pri-miR156a is important for miR156a biogenesis, with the second stem adjacent to the first cleavage site playing a role in regulating ambient temperature-responsive flowering.

## Introduction

MicroRNAs (miRNAs) are small non-coding RNAs that negatively regulate expression of their target genes via sequence- specific mRNA degradation or translational repression (Carrington and Ambros, 2003). MiRNAs originate from precursor primary miRNAs (pri-miRNAs), which have hairpin structures composed of an upper stem with a terminal loop (or loops), a miRNA/miRNA* duplex, and a lower stem. In plants, the mature miRNA from the pri-miRNA is generated by DICER-LIKE 1 (DCL1), which forms a complex with HYPONASTIC LEAVES1 (HYL1) and SERRATE (SE) for precise and efficient miRNA biogenesis (Kurihara *et al.*, 2006). The miRNA/miRNA* duplex is protected by methylation via HUA ENHANCER 1 (HEN1) (Yang *et al.*, 2006; Tsai *et al.*, 2014) and then exported to the cytoplasm. One strand of the miRNA/miRNA* duplex loads into the ARGONAUTE complex (Poulsen *et al.*, 2013). The miRNA then guides the complex to its target RNAs that have complete or partial complementarity to the miRNA sequences (Vaucheret *et al.*, 2004; Liu *et al.*, 2014).

Sequential cleavage of pri-miRNAs by DCL1 releases mature miRNAs. The pri-miRNA cleavage patterns can be classified as ‘loop-to-base’ and ‘base-to-loop’ modes, depending on the direction of processing. In ‘base-to-loop’ processing, the first cleavage occurs below the miRNA/miRNA* duplex (as seen in Arabidopsis pri-miR172a and pri-miR169a), whereas in ‘loop-to-base’ processing, the first cleavage occurs above the miRNA/miRNA* duplex (as seen in Arabidopsis pri-miR156a and pri-miR319) (Bologna *et al.*, 2009, 2013). Large-scale sequence analysis showed that the ‘base-to-loop’ mode likely occurs as the dominant mechanism in processing of Arabidopsis miRNAs (Bologna *et al.*, 2013).

The secondary structure of the pri-miRNA has important consequences for miRNA processing (Starega-Roslan *et al.*, 2011). For example, a ~15-nucleotide segment in the lower stem is essential for processing of pri-miR172a (Mateos *et al.*, 2010; Werner *et al.*, 2010), whereas the upper stem region has only a weak effect. A bulge adjacent to a cleavage site in the lower stem of pri-miR171a and the region 4–6 nucleotides below the miR390a/miR390a* duplex contributes to the efficiency and accuracy of processing of miR171a and miR390a (Cuperus *et al.*, 2010; Song *et al.*, 2010). In contrast, the conserved upper stem of pri-miR319, including a terminal loop, plays a crucial role in sequential cleavage for processing of miR319, whereas the lower stem region of pri-miR319 is dispensable (Bologna *et al.*, 2013). Thus, the structural determinants important for miRNA processing vary depending on the miRNA.

The proper timing of the transition to the flowering phase is important for reproductive success in plants. Among the environmental cues that affect the floral transition, ambient temperature has gained substantial attention because of increasing concerns about temperature fluctuations associated with climate change (Lee *et al.*, 2008, 2013; Kumar and Wigge, 2010; Kim *et al.*, 2012; Verhage *et al.*, 2014). Small changes in ambient temperature have strong effects on flowering, which can eventually disturb ecosystems and crop production (Lee *et al.*, 2007; Kang *et al.*, 2009; Woodward *et al.*, 2010). Therefore, understanding the molecular mechanism by which ambient temperature affects flowering can provide important information for predicting and mitigating the effects of climate change.

Among ambient temperature-responsive miRNAs (Lee *et al.*, 2010), miR156 and miR172 play important, but opposing roles in the control of ambient temperature-responsive phenotypes before flowering (Zhou and Wang, 2013; Kim and Ahn, 2014). At low temperatures, plants overexpressing miR156 flowered later than wild-type plants and plants expressing a target mimic of miR156 produced flowered earlier (Kim *et al.*, 2012); this occurs via miR156 regulation of the expression of *SQUAMOSA PROMOTER BINDING PROTEIN-LIKE* (*SPL*) family members (Wu and Poethig, 2006; Kim *et al.*, 2012). By contrast, plants overexpressing miR172 showed an early flowering phenotype, by targeting genes encoding AP2-like transcription factors (Aukerman and Sakai, 2003), regardless of temperature changes (Lee *et al.*, 2010). Thus, overexpression of miR156 and miR172 has opposite effects on flowering time. The structural determinants of miR172 processing have been revealed (Mateos *et al.*, 2010; Werner *et al.*, 2010), but the structural determinants of miR156 processing and their effects on flowering time at different temperatures remain unknown.

The study reported here analyzed the important structural determinants of pri-miR156a processing to produce miR156, by analyzing the effects of various mutations that perturb the secondary structure of pri-miR156a at different temperatures. It demonstrated that the stem region adjacent to the first cleavage site is important for miR156a biogenesis, and that the second stem segment in the upper stem of pri-miR156a plays a role in ambient temperature-responsive miR156a processing.

## Materials and methods

### Plant materials and measurement of leaf numbers

The Arabidopsis ecotype Columbia-0 (Col-0) was used for transformation. The plants were grown in soil at 23 °C and 16 °C under long-day (LD) conditions (16h light, 8h dark) with a light intensity of 120 μmol m^−2^s^−1^. The flowering time was measured by counting the number of primary rosette and cauline leaves of homozygous plants when the primary inflorescence had reached a height of 5cm. To isolate homozygous plants of each transgenic line, the leaf numbers of primary transformants that overexpressed each mutation were first scored in the T_1_ generation and a few representative lines that exhibited a 3:1 segregation ratio and leaf numbers close to the mean values among the T_1_ population were selected. At least 22 homozygous plants from each transgenic line were used to score leaf number values at both temperatures. The distribution of leaf numbers is presented as a box plot (Spitzer *et al.*, 2014). In our box plots, center lines show the medians; box limits indicate the 25th and 75th percentiles as determined by R software; whiskers extend 1.5 times the interquartile range (IQR) from the 25th and 75th percentiles, outliers that exceeded the 1.5× IQR are represented by dots.

### Measurement of temperature responsiveness

The leaf number ratio (LNR) (16 °C/23 °C) was used as an indicator of ambient temperature-sensitivity (Blazquez *et al.*, 2003; Lee *et al.*, 2007). The typical LNR value of wild-type Col-0 plants is ~2.0, implying that Col-0 plants produce ~2-fold more leaves at 16 °C compared with 23 °C. A completely ambient temperature-insensitive plant produces an identical total number of leaves at different temperatures; thus its LNR is 1.0. Based on this criterion, plants showing LNR values >2.0 are considered temperature-hypersensitive, whereas plants having LNR values ~1.0 are considered temperature-insensitive.

### Generation of constructs carrying structural variants and plant transformation

The constructs carrying mutations in the upper stem of pri-miR156a, lower stem of pri-miR172a, and terminal stem-swapping variants of pri-miR156a/pri-miR172a were synthesized [Cosmogenetech (Seoul, Korea)]. After sequence confirmation of individual constructs, these constructs were cloned into the pCHF3 vector containing the 35S promoter (Jarvis *et al.*, 1998). A modified floral dip method was used to introduce the resulting plasmids into wild-type Arabidopsis plants (Clough and Bent, 1998). The liquid culture of transformed *Agrobacterium tumefaciens* cells was harvested by centrifugation at 4000 ×*g* for 10min and resuspended in 5% sucrose solution to a final OD of 0.4. For infiltration, the bacterial suspension supplemented with 0.05% Silwet (LEHLE SEEDS, Silwet L-77) was applied to the shoot apex of wild-type Col-0 plants. Primary transformants were selected on Murashige Skoog (MS) media supplemented with kanamycin. Seedlings were grown at 23 °C and 16 °C for 7 and 11 d, respectively, until they reached an identical developmental stage (1.02: 2 rosette leaves>1mm) (Boyes *et al.*, 2001) and then transferred to soil for measurement of flowering time.

### Absolute quantification of primary transcripts of miR156

For absolute quantification of the levels of pri-miR156a, b, c, and d, 10-fold serial dilutions from 10^−1^ to 10^−10^ (copy number/mg tissue) were used to generate a standard curve of transcript levels of each pri-miR156 locus. To amplify pri-miR156a, b, c, and d transcripts, qPCR was performed using a LightCycler 480 (Roche, USA) and LightCycler 480 CYBR Green I Master Mix (Roche, USA), and primers specific for each miR156 locus, using cDNA produced from RNA extracted from wild-type plants grown at 23 °C or 16 °C. qPCR for both standard curve generation and amplification of pri-miR156 transcripts was performed in two biological replicates, and three technical replicates for each, with similar results. Oligonucleotide sequences are presented in Supplementary Table S1 at *JXB* online.

### 5′-RLM-RACE for cleavage site mapping

For cleavage site mapping and determination of the abundance of cleavage products, modified 5′-RLM-RACE (RNA ligase-mediated rapid amplification of 5′ cDNA ends) was performed as described previously (Llave *et al.*, 2002). Total RNA was prepared from 9-day-old seedlings with the Plant RNA Purification Reagent (Invitrogen) and was ligated to the RNA oligo-adaptor (5′-RACE adapter) with T4 RNA ligase (Ambion). The oligo-dT primer was used to prime cDNA synthesis with SuperScript III reverse transcriptase (Invitrogen, USA). PCR amplification was performed with a 5′-RACE outer primer and a gene-specific 3′ outer primer. A second round of nested PCR was done using two sets of 5′-RACE inner and gene-specific inner primers. For semi-quantitative measurements, 5′-RACE products were separated and hybridized with a probe specific to the 5′-RACE adapter sequence and *UBQ10*. For cleavage site mapping, 5′-RACE products were cloned into a T-A cloning vector (RBC) and sequenced. Oligonucleotide sequences are presented in Supplementary Table S1.

### MiRNA northern blot analysis

**Fig. 1.**
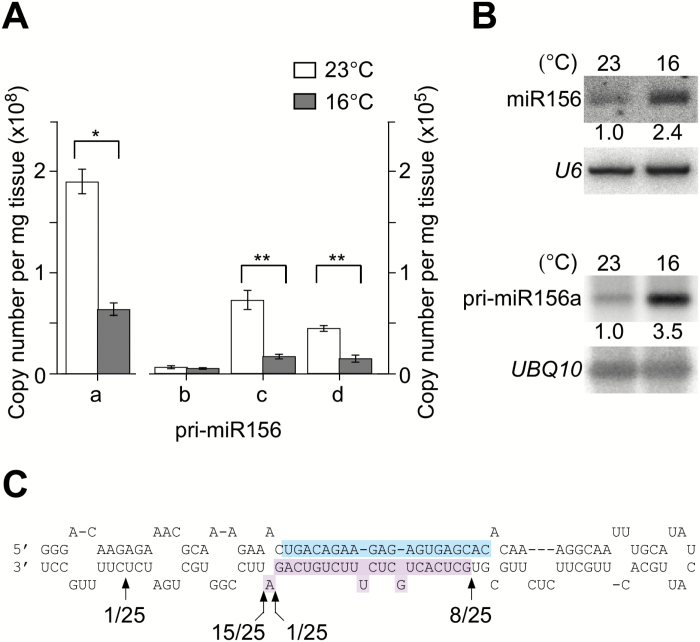
Levels of different primary transcripts of miR156 (pri-miR156) and mature miR156 at different temperatures. (A) Absolute quantification of pri-miR156a, b, c, and d transcripts in 8-day-old wild-type plants grown at 23 °C and 16 °C (*, *P*<0.01; **, *P*<0.001). (B) Levels of mature miR156 determined by northern blot analysis (upper panel) and levels of cleaved products of pri-miR156a determined by 5′-RLM-RACE (lower panel) in 8-day-old wild-type plants grown at 23 °C and 16 °C. The relative band intensities of mature miR156 and cleaved pri-miR156a are shown. *U6* and *UBQ10* were used as loading controls in each experiment. (C) Map of cleavage sites of pri-miR156a. The mature miR156a and miR156* sequences are shown in cyan and purple, respectively. The arrows indicate the end of amplicons with the fraction of sequenced clones corresponding to each site. The numbers below the arrows indicate the number of sequenced clones corresponding to each site.

These experiments used an enhanced miRNA detection method by chemical cross-linking with N-(3-Dimethylaminopropyl)-N′-ethylcarbodiimide hydrochloride (EDC) (Sigma) (Pall and Hamilton, 2008), except the miR156 blot presented in Fig. 1B, which was performed using the UV-crosslinking method (Lee *et al.*, 2010). Seven-day-old homozygous plants (23 °C) or 11-day-old homozygous plants (16 °C) of representative lines were grown and pooled to extract total RNA using Plant RNA Purification Reagent (Invitrogen). Total RNA (10 μg for the miR156 detection and 15 μg for the miR172 detection) was loaded onto 17% denaturing polyacrylamide gels containing 7M urea and electrophoresed, then transferred to Hybond-NX neutral nylon membrane (GE Healthcare), which was crosslinked with EDC. The membrane was hybridized with probes labeled at the 3′ end with [γ-^32^P] ATP using OptiKinase (USB Corp., USA). The hybridized membranes were exposed and analyzed using Fuji BAS FLA-7000 (FUJI, Japan). *U6* was used as an internal control. All miRNA northern blot experiments were performed in two biological replicates (independently harvested samples on different days) and one representative result is shown.

### qPCR analysis

For gene expression analysis, 1 μg total RNA was treated with DNaseI (New England Biolabs) and subjected to complementary DNA synthesis using the First Strand cDNA Synthesis Kit (Roche). The qPCR analysis was carried out using KAPA SYBR Green I Master Mix (KAPA Biosystems). Two stably expressed genes (*AT1G13320* and *AT2G28390*) were used as reference genes for quantification (Hong *et al.*, 2010). All qPCR experiments were performed in two biological replicates, and three technical replicates for each, with similar results. Oligonucleotide sequences are shown in Supplementary Table S1.

## Results

### MiR156 accumulation at different temperatures is regulated at the processing level

To determine the major locus of *MIR156* that produces mature miR156, an absolute quantification analysis using qPCR was performed, using primers specific to each locus. This analysis showed that *MIR156a* was the major locus that produced mature miR156 at 23 °C and 16 °C in wild-type plants (Fig. 1A; Supplementary Fig. S1). Interestingly, pri-miR156a levels were significantly higher at 23 °C than at 16 °C in 7-day-old wild-type plants. In contrast to the high levels of pri-miR156a at 23 °C, mature miR156 levels were higher at 16 °C (Fig. 1B, upper panel). We performed RNA ligase-mediated rapid amplification of 5′ cDNA ends (5′-RLM-RACE) to determine the abundance of cleavage products. It revealed that more cleavage of pri-miR156a occurred at 16 °C (Fig. 1B, lower panel), suggesting that pri-miR156a was rapidly cleaved into mature miR156 at this temperature. These observations suggested that accumulation of miR156a is regulated at the processing level.

We also used 5′-RLM-RACE to detect cleavage sites from pri-miR156a. Sequencing the resulting amplicons revealed two major products of different sizes. This implied that cleavages occurred at two distinct sites along the pri-miR156a hairpin (Fig. 1C; Supplementary Fig. S2). This also indicated that pri-miR156a followed the non-canonical ‘loop-to-base’ processing model (Bologna *et al.*, 2013).

### Effect of structural variants in the pri-miR156a upper stem on miR156 processing

To identify structural determinants that are important for pri-miR156a processing, various mutations that open a stem, close a bulge, extend a stem/bulge, or delete a stem/bulge in the upper stem of pri-miR156a were generated (Supplementary Fig. S3A, B) and analyzed the effects of overexpressing these mutant versions. The name of each variant contains the miRNA name followed by a description of the type of mutation [closing unpaired bases (CUB), disruption of base pairing (DBP), extension (EXT), and deletion (DEL)] and the mutated site (Supplementary Fig. S3B). For instance, *156-DBP-S3* indicates a mutation that disrupts base pairing in stem 3 of pri-miR156a and *156-DEL-B2* indicates a mutation that has a terminal deletion from the loop to bulge 2 of pri-miR156a. To study the *in vivo* effect of each mutation, the flowering time of homozygous plants overexpressing each mutation was analyzed (see methods).

**Fig. 2.**
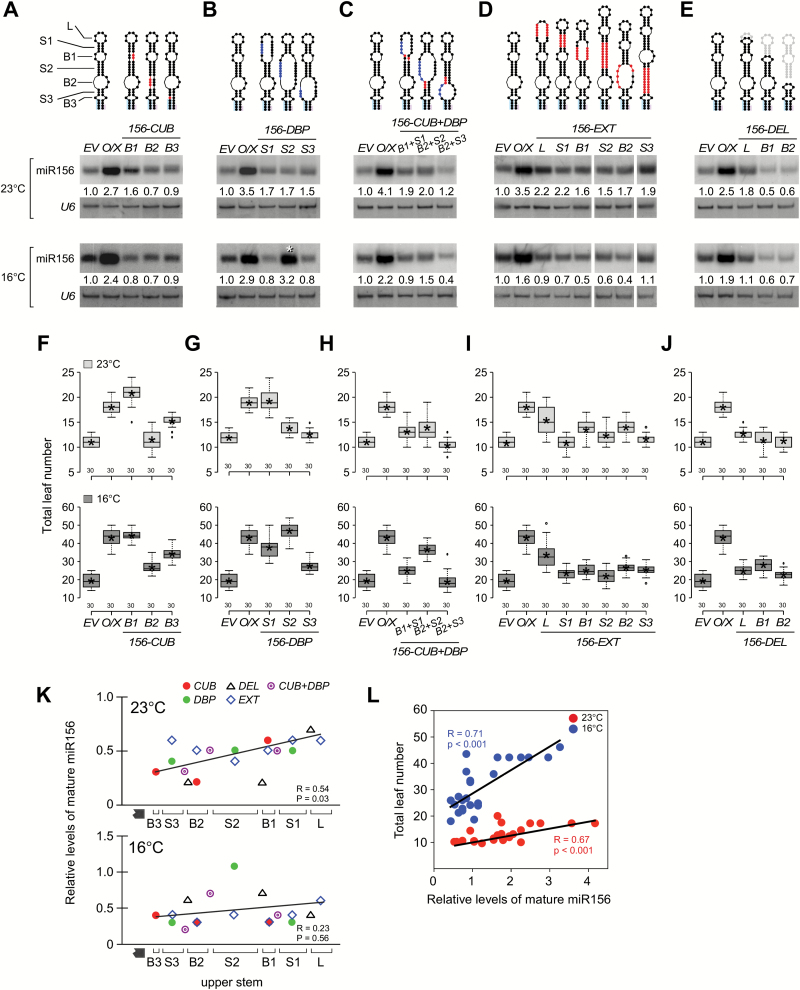
Analysis of structural variants in the upper stem of pri-miR156a. (A) Levels of mature miR156 in plants overexpressing mutations that closed unpaired bases (CUB), (B) disrupted base-pairing (DBP), (C) had combined CUB+DBP mutations, (D) had an extension (EXT), or (E) had a terminal deletion (DEL) at 23 °C and 16 °C. Mature miR156 levels of *35S::miR156a* plants (the un-mutated control) are shown in each gel blot. *U6* was used as a loading control. Introduced mutations are indicated in colors in the secondary structures of pri-miR156a above the gel blots. The numbers below each band indicate the fold-change relative to the miR156 level in *EV* plants after normalization to *U6* at each temperature. Note that miR156 levels in *DBP-S2* plants (asterisk) were similar to those of *35S::miR156a* plants at 16 °C, but not at 23 °C. (F–J) Flowering time of homozygous plants carrying each structural variation in the pri-miR156a upper stem at 23 °C and 16 °C. Distribution of leaf numbers is shown as a box plot (see methods for further information for box plots). *O/X*, plants overexpressing the un-mutated constuct (*35S∷miR156a*) (K) Relationship between mature miR156 levels and structural mutations in the upper stem of pri-miR156a. Quantification values of mature miR156 were plotted against the location of each mutation. The mature miR156 levels are normalized against the miR156 levels of un-mutated control (*35S::miR156a*) plants used in each northern blot analysis (Fig. 2A–E). A solid line indicates the trend line. Hatched box, miR156a/miR156* duplex; R, Pearson correlation coefficient. (L) Correlation between mature miR156 levels (Fig. 2A–E) and flowering time (Fig. 2F–J) of plants carrying structural variants in the pri-miR156a upper stem at 23 °C and 16 °C. R, Pearson correlation coefficient.

Northern blot analyses were performed to measure mature miR156 levels in plants overexpressing each structural variant. In RNA from these plants, a band with identical mobility to the bands detected from empty vector (*EV*) control plants and plants overexpressing the un-mutated construct (*35S::miR156a*) was detected, suggesting that these mutations did not alter the size of mature miR156. Plants overexpressing these mutations accumulated less mature miR156 at 23 °C and 16 °C, compared to the un-mutated control plants (Fig. 2A–E). For instance, although mature miR156 levels increased by 2.7-fold (relative to *EV* plants) in plants overexpressing the un-mutated construct, plants overexpressing *156-CUB-B1*, *156-CUB-B2*, and *156-CUB-B3* mutations caused only 1.6-, 0.7-, and 0.9-fold increases, respectively, at 23 °C (Fig. 2A). A lower increase in mature miR156 levels was also seen in *CUB* plants at 16 °C. This suggested that closing unpaired bases in the upper stem of pri-miR156a affected miR156 processing at both temperatures. Similarly, plants overexpressing *156-DBP-S1, 156-DBP-S2,* and *156-DBP-S3* mutations showed a lower increase in mature miR156 levels than *35S::miR156a* plants at both temperatures, except for *156-DBP-S2* at 16 °C (asterisk in Fig. 2B). The *156-DBP-S2* plants showed comparable levels of miR156 only at 16 °C. An independent line (#45-21-04; cf. #45-26-16: a reference line of *156-DBP-S2*) of *156-DBP-S2* plants also showed similar results (Supplementary Fig. S4A). The levels of target genes of miR156 (*SPL3*, *SPL4* and *SPL5*) were unaltered in both lines of *156-DBP-S2* plants at 23 °C, but down-regulated at 16 °C, like in *35S::miR156a* plants (Supplementary Fig. S4B). Plants overexpressing combined *CUB* and *DBP* mutations also showed lower miR156 levels than *35S::miR156a* plants at both temperatures (Fig. 2C). Likewise, plants overexpressing *EXT* mutations also showed lower miR156 levels than the un-mutated control plants at both temperatures (Fig. 2D), suggesting that modification of the length of any stem/bulge affected miR156 processing. Various terminal deletions were also tested. Mature miR156 levels in *156-DEL-B2* plants were lower than in *156-DEL-L* plants and *35S::miR156a* plants at both temperatures (Fig. 2E), suggesting that a longer deletion strongly reduced miR156 levels. This analysis suggested that structural variations in the upper stem, which is adjacent to the first cleavage site of pri-miR156a, affected miR156 processing at both temperatures.

An interesting observation was that mutations introduced closer to the miR156a/miR156a* duplex generally showed lower mature miR156 levels. When the positions of each mutation were plotted against the relative levels of mature miR156, an obvious decrease in the miR156 levels was observed for mutations towards the miR156/miR156* duplex, especially at 23 °C (Fig. 2K).

5′-RLM-RACE was performed to test whether these mutations affected cleavage site selection of pri-miR156a. It revealed that cleavage site selection was largely unaffected by mutations introduced in the pri-miR156a upper stem (Supplementary Fig. S5). This indicates that cleavage accuracy was not affected in structural variants in the pri-miR156a upper stem.

### Effect of structural variants in the pri-miR156a upper stem on flowering time

To test the *in vivo* effect of a mutation introduced in the upper stem of pri-miR156a, we analyzed the changes in flowering time caused by overexpression of each mutation, by scoring the number of leaves produced when the primary inflorescence had reached a height of 5cm. As overexpression of pri-miR156a caused a delay in flowering time (i.e. plants flower with more leaves) in addition to the acceleration in the leaf initiation rate (Supplementary Fig. S6) (Schwab *et al.*, 2005; Wu and Poethig, 2006; Kim *et al.*, 2012), perturbation of structures required for miR156 processing should cause plants to flower earlier (i.e. with fewer leaves) than un-mutated control plants. Analysis of flowering time showed that, regardless of the mutation type, at 23 °C almost all transgenic plants flowered earlier compared with the control plants (Fig. 2F–J; Table 1; Supplementary Figs S6, 7A–E), consistent with the reduced miR156 levels in the plants expressing the mutated constructs. The differences were statistically significant (Supplementary Fig. S8). For instance, *156-DBP-S2* (12.9 leaves), and *156-DBP-S3* plants (11.7 leaves) flowered earlier than *35S::miR156a* plants at 23 °C. Similarly, almost all *EXT* plants flowered earlier than *35S::miR156a* plants. In addition, *156-DEL-L* (12.7 leaves) and *156-DEL-B2* plants (11.3 leaves) also flowered earlier, compared to *35S::miR156a* plants. At 16 °C, almost all transgenic lines flowered earlier than *35S::miR156a* plants regardless of the mutation type, as at 23 °C (Fig. 2F–J; Table 1). Taking these observations together, our analysis indicated that mutations introduced in the upper stem of pri-miR156a caused visible changes in flowering time.

**Table 1.** Flowering time of plants expressing structural variants of pri-miRNAs used in this study

Pri-miRNA	Mutation type	Variant name	23 °C			16 °C			LNR (16 °C /23 °C)	*n* (23 °C, 16 °C)
			Rosette	Cauline	Total	Rosette	Cauline	Total		
Pri-miR156a	Control	*EV*	8.7	2.3	11.0	15.5	3.9	19.4	1.8	30, 30
		*35S::miR156a*	15.2	2.8	18.0	36.0	7.0	43.0	2.4	30, 30
	Closing unpaired bases	*156-CUB-B1*	17.5	3.3	20.8	37.9	6.4	44.3	2.1	30, 30
		*156-CUB-B2*	9.3	2.2	11.5	21.5	5.3	26.8	2.3	30, 30
		*156-CUB-B3*	12.8	2.4	15.2	28.4	5.8	34.2	2.3	30, 30
	Disrupting base-pairing	*156-DBP-S1*	15.2	3.1	18.3	28.7	8.9	37.6	2.1	30, 30
		*156-DBP-S2*	11.0	1.9	12.9	41.3	5.6	46.9	3.6	30, 30
		*156-DBP-S3*	9.7	2.0	11.7	21.7	5.8	27.5	2.4	30, 30
	CUB+DBP	*156-CUB- B1*+*DBP-S1*	10.9	2.2	13.1	20.3	4.6	24.9	1.9	30, 30
		*156-CUB- B2*+*DBP-S2*	11.7	2.2	13.9	30.0	6.7	36.7	2.6	30, 30
		*156-CUB- B2*+*DBP-S3*	8.1	2.3	10.4	15.0	3.8	18.8	1.8	30, 30
	Extension	*156-EXT-L*	13.3	2.1	15.4	28.4	5.2	33.6	2.2	30, 30
		*156-EXT-S1*	8.9	1.9	10.8	18.3	5.3	23.6	2.2	30, 30
		*156-EXT-B1*	10.9	2.6	13.5	20.1	5.0	25.1	1.9	30, 30
		*156-EXT-S2*	10.2	2.1	12.3	18.0	4.0	22.0	1.8	30, 30
		*156-EXT-B2*	11.2	2.8	14.0	21.9	4.8	26.7	1.9	30, 30
		*156-EXT-S3*	9.1	2.5	11.6	20.2	5.4	25.6	2.2	30, 30
		*156-DEL-L*	10.3	2.4	12.7	19.6	5.1	24.7	1.9	30, 30
	Terminal deletion	*156-DEL-B1*	8.5	2.9	11.4	21.5	6.6	28.1	2.5	30, 30
		*156-DEL-B2*	8.6	2.7	11.3	17.6	5.2	22.8	2.0	30, 30
Pri-miR172a	Control	*EV*	8.9	2.1	11.0	16.6	3.6	20.2	1.8	30, 30
		*35S::miR172a*	2.3	3.7	6.0	3.5	2.8	6.3	1.1	30, 24
	Closing unpaired bases	*172-CUB-B6*	6.9	1.9	8.8	9.8	1.6	11.4	1.3	33, 34
		*172-CUB-B7*	10.3	2.0	12.3	20.2	3.3	23.5	1.9	24, 24
		*172-CUB-B8*	3.7	1.8	5.5	5.4	1.9	7.3	1.3	22, 24
	Disrupting base-pairing	*172-DBP-S6*	6.7	2.3	9.0	14.6	4.2	18.8	2.1	26, 35
		*172-DBP-S7*	10.6	2.5	13.1	25.2	6.8	32.0	2.4	36, 36
		*172-DBP-S8*	7.4	1.7	9.1	7.9	1.5	9.4	1.0	36, 24
		*EV*	8.7	2.3	11.0	15.5	3.9	19.4	1.8	30, 30
Pri-miR156a & Pri-miR172a	Control	*35S::miR156a*	15.2	2.8	18.0	36.0	7.0	43.0	2.4	30, 30
		*35S::miR172a*	2.3	3.7	6.0	2.9	3.3	6.2	1.0	30, 30
	Stem-swapping	*LS156DX/172US*	8.1	2.4	10.5	14.0	3.3	17.3	1.6	30, 30
		*LS172DX/156US*	4.3	2.1	6.4	2.9	3.5	6.4	1.0	30, 30

*35S::miR156a*: wild-type plants overexpressing pri-miR156a, *35S::miR172a*: wild-type plants overexpressing pri-miR172a, B: bulge, DX: miRNA/miRNA* duplex, *EV*: wild-type plants overexpressing the empty vector, LNR: leaf number ratio (16 °C/23 °C), LS: lower stem, S: stem, US: upper stem, *n*: number of plants counted.

Correlation analysis between flowering time and mature miR156 levels in transgenic lines extended this observation. Pearson correlation coefficient values of transgenic plants grown at 23 °C and 16 °C were 0.67 (*P*<0.001) and 0.71 (*P*<0.001), respectively (Fig. 2L). This indicated a significant positive correlation between flowering time and mature miR156 levels in transgenic plants tested at both temperatures.

### S2, the second stem adjacent to the first cleavage site of pri-miR156a, is important for the temperature response of miR156a

Almost all of the transgenic plants showed reduced levels of mature miR156 at both temperatures compared to the un-mutated control plants, with the exception of plants carrying *156-DBP-S2*, in which opening S2 caused a large bulge along with B1 and B2 (Fig. 2B). Mature miR156 levels in *156-DBP-S2* plants were reduced only at 23 °C, but not at 16 °C (Fig. 2B; Table 1). This suggested that the *156-DBP-S2* mutation did not affect miR156 biogenesis at 16 °C.

**Fig. 3.**
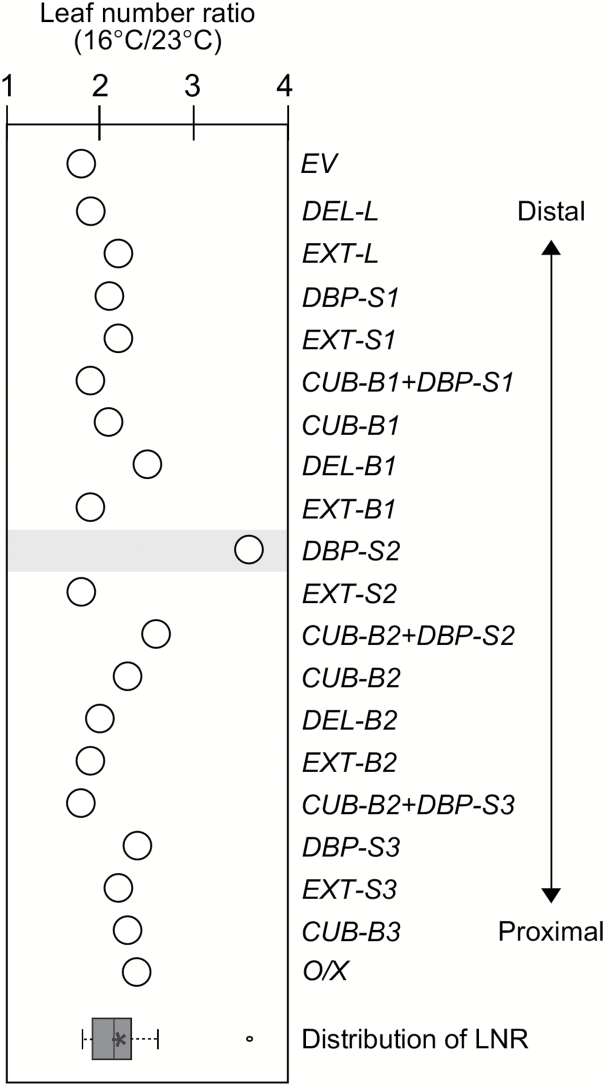
Leaf number ratio (16 °C/23 °C) of plants overexpressing structural variants of pri-miR156a. Distribution of LNR of the plants shown in this graph is shown as a box plot at the bottom of graph (see methods for further information on box plots). *O/X*, plants overexpressing the un-mutated constuct (*35S∷miR156a*). Note that *156-DBP-S2* plants showed hypersensitivity to ambient temperature changes.

Next, measurements of the leaf number ratio (LNR, number of leaves at flowering at 16 °C/number of leaves at 23 °C) (Kim *et al.*, 2012) tested whether the different levels of mature miR156 seen in *156-DBP-S2* plants caused different phenotypic responses to ambient temperature. The LNR values of *EV* plants and *35S::miR156a* plants were 1.8 and 2.4, respectively, indicating that miR156a overexpression caused changes in flowering time that responded sensitively to changes in ambient temperature (Fig. 3; Table 1). The LNR of almost all transgenic plants (average value=2.2) was lower than that of *35S::miR156a* plants, indicating that mutations in the pri-miR156a upper stem caused decreased sensitivity to differences in ambient temperature. Interestingly, *156-DBP-S2* plants showed an LNR value of 3.6, suggesting that the *156-DBP-S2* mutation caused dramatic hypersensitivity to ambient temperature (Fig. 3; Table 1). An independent line (#45-21-04) of *156-DBP-S2* plants also showed similar hypersensitivity (Supplementary Fig. S4C, D), confirming the notion that opening S2 caused hypersensitivity to ambient temperature. By contrast, the LNR value of *156-EXT-S2* plants was lower (1.8) than that of *35S::miR156a* plants, indicating that extending S2 caused an opposite effect (Fig. 3; Table 1). *156-CUB-B2/156-DBP-S2* plants, which had combined mutations of *156-CUB-B2* and *156-DBP-S2*, showed an intermediate LNR value (2.6) between *156-DBP-S2* (3.6) and *156-EXT-S2* plants (1.8) (Fig. 3; Table 1; Supplementary Fig. S7H). These observations suggested that S2 plays a role in miR156 processing at different ambient temperatures.

### Effect of structural variants in the pri-miR172a lower stem on miR172 processing

The above observations showed that the upper stem of pri-miR156a, the region adjacent to the first cleavage site, is important for miR156 processing. To determine whether the observations from pri-miR156a might apply to other miRNAs, the next experiments examined pri-miR172a, an important ambient temperature-responsive miRNA that follows a canonical ‘base-to-loop’ processing mode (Bologna *et al.*, 2013; Mathieu *et al.*, 2009), and analyzed the effects of various mutations in the lower stem of pri-miR172a on miR172 processing and leaf numbers (Supplementary Fig. S3C, D).

**Fig. 4.**
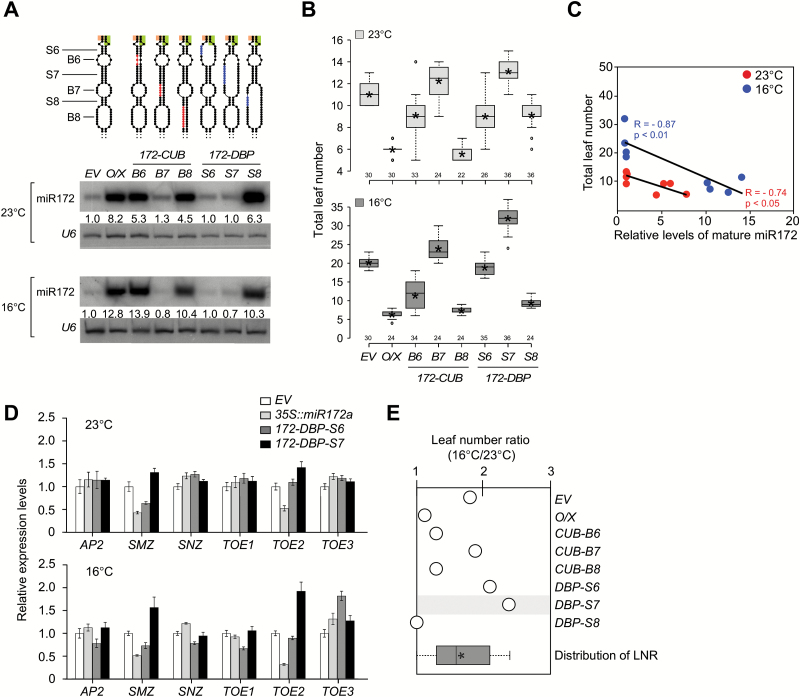
Analysis of structural variants in the lower stem of pri-miR172a. (A) Levels of mature miR172 in plants overexpressing mutations grown at 23 °C and 16 °C. *U6* was used as a loading control. Introduced mutations are indicated in colors in the secondary structures of pri-miR156a above the gel blots. The numbers below each band indicate the fold-change relative to the miR172 level in *EV* plants after normalization to *U6* at each temperature. (B) Flowering time of homozygous plants carrying each structural variant in the pri-miR172a upper stem at 23 °C and 16 °C. Distribution of leaf numbers is shown as a box plot (see methods for further information for box plots). (C) Correlation between mature miR172 levels (Fig. 4A) and flowering time (Fig. 4B) in plants carrying structural variants in the pri-miR172a lower stem at 23 °C and 16 °C. R, Pearson correlation coefficient. (D) Expression levels of miR172 target genes in 9-day-old seedlings of *172-DBP-S6* and *172-DBP-S7* plants at 23 °C and 16 °C, determined via qPCR. Error bars indicate standard deviation. Expression levels of each gene in *EV* plants were set to 1. *AP2*, *APETALA2*; *SMZ*, *SCHLAFMÜTZE*; *SNZ*, *SCHNARCHZAPFEN*, ; *TOE1, 2, 3*, *TARGET OF EAT1, 2, 3* (E) Leaf number ratio (16 °C/23 °C) of plants overexpressing structural variants of pri-miR172a. Distribution of LNR of the plants presented in this graph is shown as a box plot at the bottom of graph. Note that *172-DBP-S7* plants showed slightly increased sensitivity to ambient temperature changes. *O/X*, plants overexpressing the un-mutated constuct (*35S∷miR172a*).

Small RNA northern blot analyses showed that among *CUB* variants, *172-CUB-B7* plants showed dramatically reduced levels of mature miR172, whereas *172-CUB-B6* and *172-CUB-B8* plants still showed high levels of mature miR172, which were comparable to those seen in the control plants expressing the un-mutated construct (*35S::miR172a*) at both temperatures. This suggested that closing B7 severely affected miR172 processing (Fig. 4A). Among *DBP* variants, *172-DBP-S6* and *172-DBP-S7* plants showed dramatically reduced miR172 levels at both temperatures, whereas *172-DBP-S8* plants still showed high levels of mature miR172 (Fig. 4A). These results suggested that *172-CUB-B7, 172-DBP-S6*, and *172-DBP-S7* mutations interfered with miR172 processing at both temperatures.

### Effect of structural variants in the pri-miR172a lower stem on leaf numbers

The differences in mature miR172 levels caused by structural variations in the pri-miR172a lower stem were largely consistent with their effects on flowering time. Overexpression of miR172 caused acceleration of flowering (i.e. plants flowered with fewer leaves) (Supplementary Fig. S6); therefore, perturbation of structures required for miR172 processing should cause delayed flowering (i.e. an *EV*-like phenotype) compared with plants expressing the un-mutated control (*35S::miR172a*). At 23 °C, *172-CUB-B7* and *172-DBP-S7* plants showed similar flowering time (12.3 and 13.1 leaves, respectively) to *EV* plants, suggesting that these mutations severely affected miR172 function. In contrast, *172-CUB-B6*, *172-CUB-B8, 172-DBP-S6*, and *172-DBP-S8* plants flowered earlier (8.8, 5.5, 9.0, and 9.1 leaves, respectively), similar to un-mutated control plants (Fig. 4B; Table 1; Supplementary Fig. S7F). A similar effect on flowering time was also seen at 16 °C. This indicated that perturbation of the structure of a region including B7 and S7 caused a visible phenotype. Correlation analysis showed significant negative correlation between flowering time and mature miR172 levels in transgenic plants tested at both temperatures, with Pearson correlation coefficient values of transgenic plants grown at 23 °C and 16 °C being −0.74 (*P*<0.05) and −0.87 (*P*<0.01), respectively (Fig. 4C).

### S7, the second stem adjacent to the first cleavage site of pri-miR172a, may be important for the temperature response of miR172a

Among flowering time data, one notable observation was that *172-DBP-S7* plants flowered even later (13.1 and 32.0 leaves at 23 °C and 16 °C, respectively) than *EV* plants at both temperatures (Fig. 4B; Table 1). Consistent with the delayed flowering phenotype of *172-DBP-S7* plants, the transcript levels of *TARGET OF EARLY ACTIVATION TAGGED 2* (*TOE2*) and *SCHLAFMÜZE* (*SMZ*), a floral repressor targeted by miR172 (Aukerman and Sakai, 2003; Lee *et al.*, 2010), increased in *172-DBP-S7* plants at both temperatures (Fig. 4D). We also analyzed miR156 levels because miR172 is known to act downstream of the miR156-*SPL* module for flowering time regulation (Wu *et al.*, 2009). We found that miR156a levels increased and *SPL9* and *SPL10* decreased in *172-DBP-S7* plants at 16 °C (Supplementary Fig. S9A, B). Considering that mature miR172 levels were not dramatically lower than those of *EV* plants, it is likely that the increased miR156a levels observed in *172-DBP-S7* plants at 16 °C are responsible for the increase in the leaf numbers at 16 °C.

LNR measurement revealed that *172-DBP-S7* plants showed slightly increased sensitivity to temperature (LNR: 2.4) compared to *EV* plants, whereas *172-CUB-B7* (LNR: 1.9) and *172-DBP-S6* (LNR: 2.1) plants showed similar temperature responses to *EV* plants (LNR: 1.8), suggesting that S7 is important for the temperature response. By contrast, *172-CUB-B6*, *172-CUB-B8*, and *172-DBP-S8* plants, in which mature miR172 production was unaffected, still showed temperature-insensitive changes in flowering time (LNR: 1.3, 1.3, and 1.0, respectively), like plants expressing the un-mutated control construct (*35S::miR172a*) (Fig. 4E; Table 1). The increased sensitivity in temperature-responsive flowering of *172-DBP-S7* plants was also observed in the T_1_ generation (Supplementary Fig. S7I). Our analysis of plants overexpressing constructs with structural variations in the pri-miR172a lower stem suggested that the second stem (S7) adjacent to the first cleavage site plays a role in miR172 processing and temperature-dependent flowering (Werner *et al.*, 2010), as seen in S2 in the upper stem of pri-miR156a (Figs 2B, G, 3).

### Effects of swapping the terminal stem of pri-miR156a on miR156 processing and leaf numbers

**Fig. 5.**
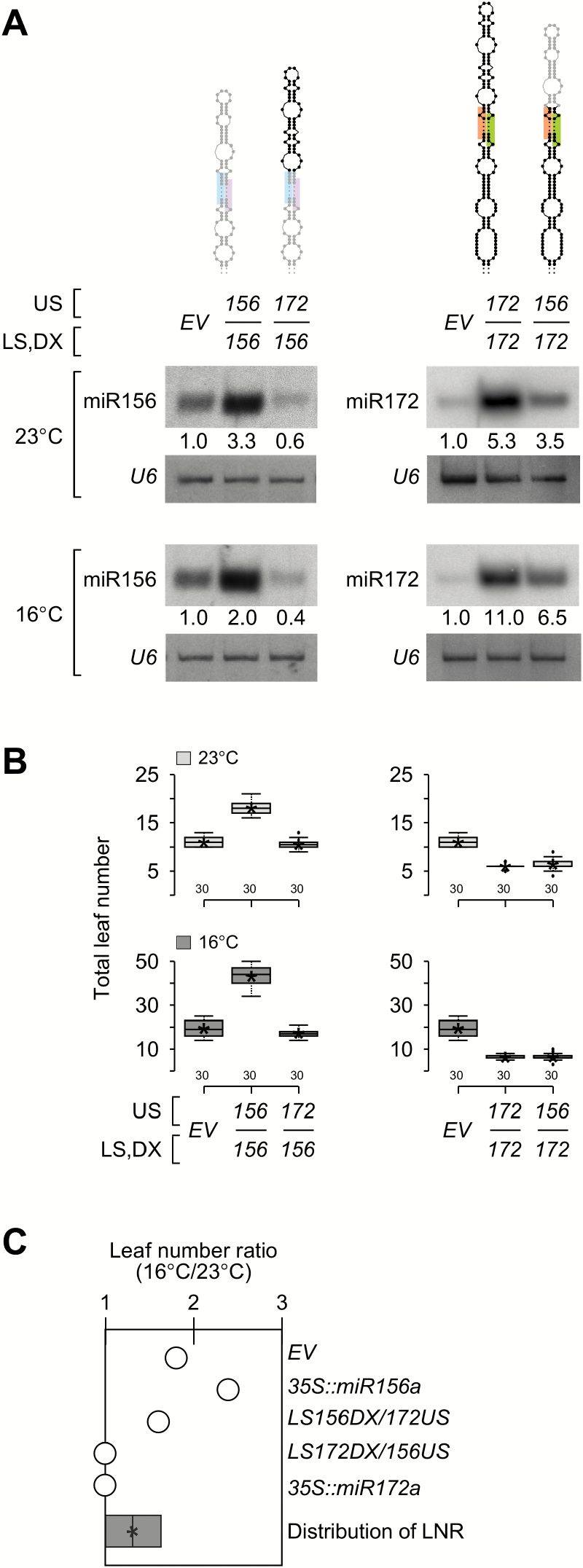
Analysis of stem-swapping variants of pri-miR156a and pri-miR172a. (A) A diagram of pri-miR156a and pri-miR172a swapping variants (upper panel) and levels of mature miR156 and miR172 in plants carrying stem-swapping variants of pri-miR156a and pri-miR172a at 23 °C and 16 °C under LD conditions (lower panels). Sequences of pri-miR156a and pri-miR172a are shown in gray and black, respectively. *U6* was used as a loading control. The numbers below each band indicate the fold change relative to the miR156 or miR172 level in *EV* plants after normalization to *U6* at each temperature. (B) Flowering time of homozygous plants carrying stem-swapping variants of pri-miR156a and pri-miR172a at 23 °C and 16 °C. Distribution of leaf numbers is shown as a box plot (see methods for further information on box plots). (C) Leaf number ratio (16 °C/23 °C) of plants carrying stem-swapping variants of pri-miR156a and pri-miR172a. Distribution of LNR of the plants presented in this graph is shown as a box plot at the bottom of graph. Note that temperature sensitivity was greatly affected by replacing the upper stem of pri-miR156a in *LS156DX/172US* plants.

The mutational analyses of pri-miR156a and pri-miR172a suggested that the stem region adjacent to the first cleavage site is important (Figs 2, 4). To further test this notion, the effects of stem-swapping variants between pri-miR156a and pri-miR172a were tested (Fig. 5). Small RNA northern blot analyses revealed that mature miR156 levels were dramatically reduced in *LS156DX/172US* plants (Fig. 5A, left panel), which carried the lower stem of pri-miR156a and the miR156a/miR156a* duplex fused with the upper stem of pri-miR172a, compared to those of the un-mutated (*35S::miR156a*) control plants at both temperatures. However, high levels of mature miR172 were still seen in *LS172DX/156US* plants (Fig. 5A, right panel), which carried the lower stem of pri-miR172a and the miR172a/miR172a* duplex fused with the upper stem of pri-miR156a, although the level was lower than that of the control plants expressing the un-mutated pri-miR172a. This suggested that replacing the upper stem of pri-miR156a with that of pri-miR172a strongly affected miR156a processing, whereas replacing the upper stem of pri-miR172a with that of pri-miR156a did not severely affect miR172a processing.

Next, analysis of the flowering time changes caused by terminal stem-swapping variants showed that the miR156 and miR172 levels of stem-swapping variants were consistent with their changes in flowering time (Fig. 5B; Supplementary Fig. S7G). At both temperatures, *LS156DX/172US* plants showed similar flowering phenotypes (10.5 and 17.3 leaves) to *EV* plants (11.0 and 19.4 leaves) (Fig. 5B; Table 1), indicating that the severe phenotype caused by miR156 largely disappeared upon the introduction of the upper stem of pri-miR172a. By contrast, *LS172DX/156US* plants still showed dramatically early flowering (6.4 and 6.4 leaves), as seen in un-mutated *35S::miR172a* plants (6.0 and 6.2 leaves) at both temperatures, indicating that replacing the terminal stem of pri-miR172a failed to cause a visible change.

The LNR value of *LS156DX/172US* plants (1.6) indicated that the enhanced response to temperature was not seen, compared to *35S::miR156* plants (LNR: 2.4), confirming the diminished phenotypic severity by the replacement of the upper stem in pri-miR156a. In contrast, the LNR value of *LS172DX/156US* plants (1.0) indicated that *LS172DX/156US* plants still showed temperature-insensitive flowering, like *35S::miR172a* plants (Fig. 5C). These results suggested that the stem region adjacent to the first cleavage site is important for miRNA processing and ambient temperature-dependent flowering.

## Discussion

Among ambient temperature-responsive miRNAs, miR156 and miR172 have important functions in regulating temperature responses (Lee *et al.*, 2010). Interestingly, the levels of pri-miR156 and mature miR156 showed opposite patterns at different temperatures (Fig. 1), suggesting that ambient temperature regulates miR156 at the processing level, whereas miR172 seems to be regulated at both the transcriptional and processing levels (Cho *et al.*, 2012; Jung *et al.*, 2012). This study analyzed the effect of modification of pri-miR156a secondary structure to identify structural determinants important for miR156 processing.

### The important role of the pri-miR156a upper stem in miR156a processing

This study showed that the upper stem of pri-miR156a is important for miR156a processing. Almost all variants at the upper stem of pri-miR156a inhibited miR156a processing and variants closer to the miR156a/miR156a* duplex were especially effective (Fig. 2A–E). Consistent with this notion, replacing the upper stem of pri-miR156a with the upper stem of pri-miR172a abolished the effect of miR156 (Fig. 5A, B). By contrast, in the case of pri-miR172a, which is processed by a canonical ‘base-to-loop’ mechanism (Bologna *et al.*, 2013), the lower stem is important. Modification of S7 and B7 in the lower stem affected miR172 function (Fig. 4A, B) and modification of S7 in the lower stem changed temperature-dependent changes in flowering time (Fig. 4E). Replacing the upper stem of pri-miR172a with the upper stem of pri-miR156a did not affect miR172 accumulation (Fig. 5A). These results are consistent with the finding that most of the structural variants adjacent to the loop did not affect miR172 levels (Mateos *et al.*, 2010). Considering that pri-miR156a and pri-miR172a are processed by a non-canonical ‘loop-to-base’ mechanism and a canonical ‘base-to-loop’ mechanism (Fig. 1) (Bologna *et al.*, 2013), respectively, the data presented here strongly suggest that the region adjacent to the first cleavage site is critical.

Interestingly, miR156 levels even lower than those in *EV* plants were observed in some transgenic plants (for instance, *156-DEL-B1* and *LS156DX/172US* plants) (Figs 2A–E, 5A). A possible explanation for the low miR156 levels may be that miR156* levels aberrantly increased in the transgenic lines, thus leading to miR156/miR156* duplex formation to tether mature miR156. This may cause the repression/reduction of miR156 activity. Alternatively, a target gene-miRNA autoregulatory feedback loop may be involved. Such a scenario has been proposed in breast cancer cells (Lin *et al.*, 2011). MiR206 levels were dependent on expression of its target gene, *Krüppel-like factor 4*, implying that repressed expression of a target gene by an exogenous miRNA caused an inhibition of miR206 activity. Further investigation is needed to examine these hypotheses.

### The role of the second stem segment of pri-miR156a in ambient temperature-responsive miR156 processing

The results described above show that the second stem segment near the first cleavage site in pri-miR156 and pri-miR172a has a special role in the response to ambient temperature. The transgenic plants that expressed a pri-miR156a disrupted in stem 2 segment (*156-DBP-S2*), showed reduced mature miR156a levels and flowered earlier at 23 °C (Fig. 2B, G), but exhibited similar mature miR156 levels and an unaltered flowering time phenotype at 16 °C, resulting in flowering hypersensitive to changes in ambient temperature (Fig. 3). This notion is also supported by observation of the effect of structural variants on pri-miR172a. A disruption mutation in the second stem near the first cleavage site of pri-miR172a (*172-DBP-S7*) induced a similar effect to that caused by the *156-DBP-S2* mutation. The processing of miR172 in *172-DBP-S7* plants decreased more at 16 °C than at 23 °C and this caused slightly increased sensitivity to ambient temperature (Fig. 4). Our results suggest that the second stem of the pri-miR156a upper stem has a role in temperature-responsive flowering. Furthermore, disruption of the structure of stem 2 segment caused a decrease in miR156a levels only at 23 °C, indicating that the stem 2 segment of pri-miR156a mainly affects miR156 production at 23 °C, but not at 16 °C. Thus, we propose that the stem 2 of miR156 could be a potentially useful target region for the modulation of the temperature response in agricultural applications (Xia *et al.*, 2012; Yang *et al.*, 2013).

The junction of single-stranded and double-stranded RNA 15 nucleotides below the miR172a/miR172a* duplex is required for accurate miR172a processing (Mateos *et al.*, 2010; Werner *et al.*, 2010). The base-pairing properties within a specific region of miR390a precursor are also important for efficiency and accuracy of miR390a precursor processing (Cuperus *et al.*, 2010). The G-to-A substitution of the fourth base below the miR390a/miR390a* duplex of the miR390a precursor, which closed an unpaired base and affected the entropy of base pairing, caused inefficient and inaccurate miR390a processing, possibly due to the disturbance of interaction with DCL1-SE-HYL1 complex. In our study, the *172-DBP-S7* mutation disturbed the junction of single-stranded and double-stranded RNA 15 nucleotides from the miR172/miR172* duplex and resulted in increased sensitivity in temperature-responsive flowering. However, the *172-CUB-B7* mutation did not cause any apparent effect (Fig. 4E), although it also disrupted the junction. Thus, it seems likely that the second stem adjacent to the first cleavage site from the miR/miR* duplex (S2 in pri-miR156a and S7 in pri-miR172a), rather than the junction that is 15-nucleotides from the duplex, is more important for the response to ambient temperature.

An interesting question would be whether the importance of stem 2 near the first cleavage site is conserved in ambient temperature-responsive miRNAs, especially in miR156. It is difficult to answer the question because the same mature miR156 is produced from pri-miR156a-f (Lee *et al.*, 2010), despite the different sequences and structures of the upper stem region of these pri-miR156 genes (Supplementary Fig. S10). One notable example is pri-miR156c, the second most-abundant transcript that produces mature miR156 (Fig. 1A). Pri-miR156c and pri-miR156a have very similar sequences and structures. For example, stem 2 and the miR156/miR156* duplex of both pri-miR156a and pri-miR156c are 6 nucleotides apart and their stem 2 sequences are 5 and 6 nucleotides long, respectively, which is longer than those of other pri-miR156 genes. Thus, based on these similarities, it is tempting to speculate that the importance of stem 2 is conserved in pri-miR156a and pri-miR156c, two major loci producing miR156 in Arabidopsis. Further research, including swapping/mutating stem 2 between pri-miR156a and pri-miR156c, will be necessary to determine whether the role of the second segment of pri-miR156a on temperature response is conserved in pri-miR156c.

**Fig. 6.**
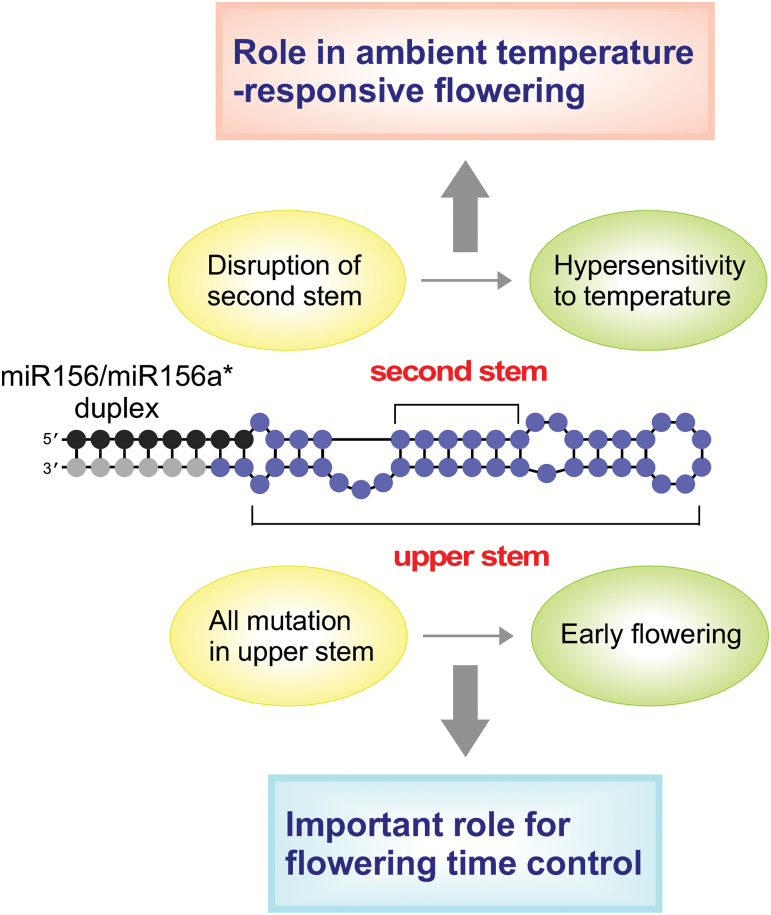
Schematic illustration showing potential structural determinants of miR156a precursor processing in the regulation of ambient temperature-responsive flowering. All mutations introduced in the upper stem of pri-miR156a affected miR156a processing and caused flowering earlier than the un-mutated control plants. Disruption of stem 2 (S2) caused flowering to be hypersensitive to the changes in ambient temperature.

In this study, we elucidated the structural features of pri- miRNAs in miRNA processing and ambient temperature-dependent changes in flowering time. This identified an important role for the upper stems of pri-miR156a and a role for the second stem of the miRNA/miRNA* duplex of pri-miR156a and pri-miR172a on the stem region adjacent to the first cleavage site for miRNA processing and ambient temperature-dependent flowering (Fig. 6). These findings provide a clue to help decode the information embedded in the sequence of the pri-miRNA in ambient temperature-responsive processing of miRNAs.

## Supplementary data

Supplementary data are available at *JXB* online.

Figure S1. Standard curves used for absolute quantification of the four loci of pri-miR156 (a, b, c, and d) and PCR efficiency of qPCR primers used for absolute quantification.

Figure S2. Schematic illustration of 5′-RLM-RACE strategy for mapping intermediates of miRNA processing.

Figure S3. Schematic illustration of structural variants of pri-miR156a and pri-miR172a.

Figure S4. Analysis of an independent line (#45-21-04) of *156-DBP-S2* plants.

Figure S5. Map of cleavage sites of structural variants in the pri-miR156a upper stem detected by 5′-RLM-RACE.

Figure S6. Flowering time phenotypes of plants overexpressing pri-miR156a upper stem variants at 23 °C and at 16 °C.

Figure S7. Leaf numbers at flowering and leaf number ratio of plants expressing structural variants of pri-miRNAs in the T_1_ generation.

Figure S8. Student’s *t*-test values between leaf numbers of mutant lines used in this study.

Figure S9. Analysis of *172-DBP-S7* plants.

Figure S10. Comparison of sequence and structure of different *MIR156* genes.

Table S1. Oligonucleotide sequences used in this study.

Supplementary Data
