# Supplementary material for: Structural determinants of miR156a precursor processing in temperature-responsive flowering in Arabidopsis
Source: J Exp Bot. 2016 Jun 21;67(15):4659–70. doi: 10.1093/jxb/erw248 (PMC4973740; doi:10.1093/jxb/erw248)
Supplement: Supplementary Data [file supp_67_15_4659__index.html]

Structural determinants of miR156a precursor processing in temperature-responsive flowering in Arabidopsis — Structural determinants of miR156a precursor processing in temperature-responsive flowering in Arabidopsis — Supplementary Data 

# Structural determinants of miR156a precursor processing in temperature-responsive flowering in Arabidopsis

## Supplementary Data

Data files

- supplementary\_figures\_S1\_S10\_table\_S1.pdf - Supplementary Data
