# Supplementary material for: Structural determinants of miR156a precursor processing in temperature-responsive flowering in Arabidopsis
Source: J Exp Bot. 2016 Jun 21;67(15):4659–70. doi: 10.1093/jxb/erw248 (PMC4973740; doi:10.1093/jxb/erw248)
Supplement: Supplementary Data [file supp_erw248_supplementary_figures_S1_S10_table_S1.pdf]

SUPPLEMENTARY DATA

Table of contents

Supplementary Fig. S1. Standard curves used for absolute quantification of the four loci of pri-miR156 (a, b, c, and d) and PCR efficiency of qPCR primers used for absolute quantification

Supplementary Fig. S2. Schematic illustration of 5′-RLM-RACE strategy for mapping intermediates of miRNA processing.

Supplementary Fig. S3. Schematic illustration of structural variants of pri-miR156a and pri-miR172a.

Supplementary Fig. S4. Analysis of an independent line (#45-21-04) of *156-DBP-S2* plants.

Supplementary Fig. S5. Map of cleavage sites of structural variants in the pri-miR156a upper stem detected by 5′-RLM-RACE.

Supplementary Fig. S6. Flowering time phenotypes of plants overexpressing pri-miR156a upper stem variants at 23°C and at 16°C.

Supplementary Fig. S7. Leaf numbers at flowering and leaf number ratio of plants expressing structural variants of pri-miRNAs in the T_1 generation.

Supplementary Fig. S8. Student`s *t*-test values between leaf numbers of mutant lines used in this study.

Supplementary Fig. S9. Analysis of *172-DBP-S7* plants.

Supplementary Fig. S10. Comparison of sequence and structure of different *MIR156* genes.

A

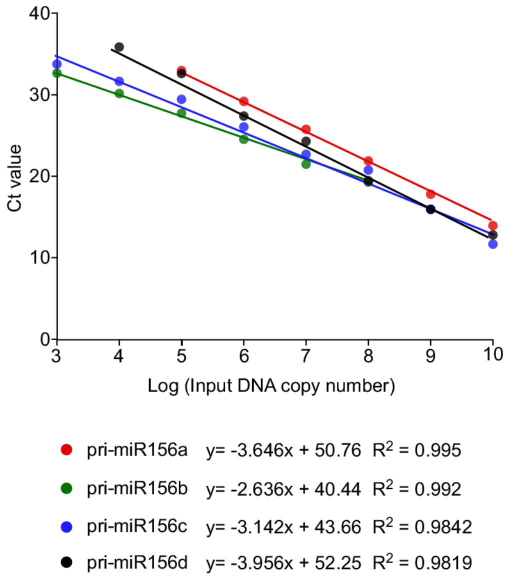

B

miR156 loci	pri-miR156a	pri-miR156b	pri-miR156c	pri-miR156d
Primer set used for qPCR	JH5241/JH5242	JH5244/JH5245	JH5248/JH5249	JH5252/JH5253
PCR efficiency	1.935	1.901	1.917	1.856

Supplementary Fig. S1. Standard curves used for absolute quantification of the four loci of pri-miR156 (a, b, c, and d) and PCR efficiency of qPCR primers used for absolute quantification.

(A) Standard curves showing the Ct values plotted versus the known concentration of serial dilutions of each pri-miR156 transcript (log scale). Note that all the standard curves overlap, indicating that our primers had similar PCR efficiency.

(B) The PCR efficiency values of primers sets used for amplification each pri-miR156 locus, calculated by using LinRegPCR (Ramakers *et al.*, 2003).

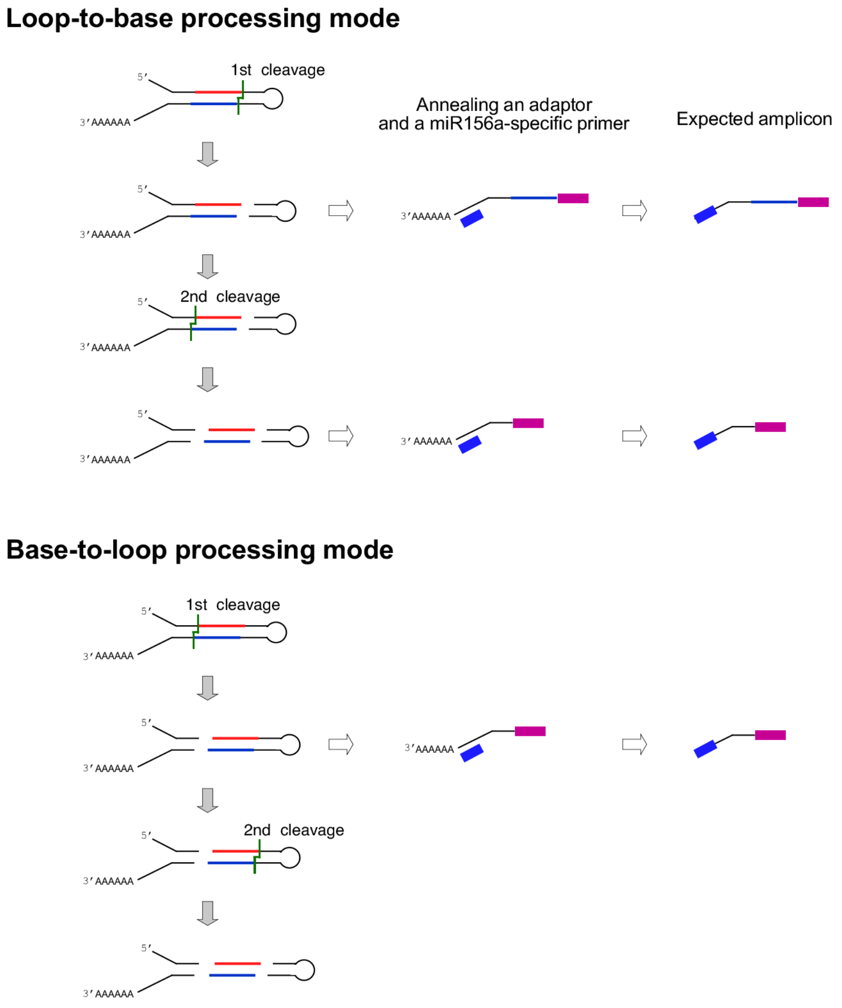

Supplementary Fig. S2. Scheme illustration of 5′-RLM-RACE strategy for mapping intermediates of miRNA processing. Note that the number of expected amplicons depends on the direction of miRNA precursor processing (loop-to-base: 2 ; base-to-loop: 1)

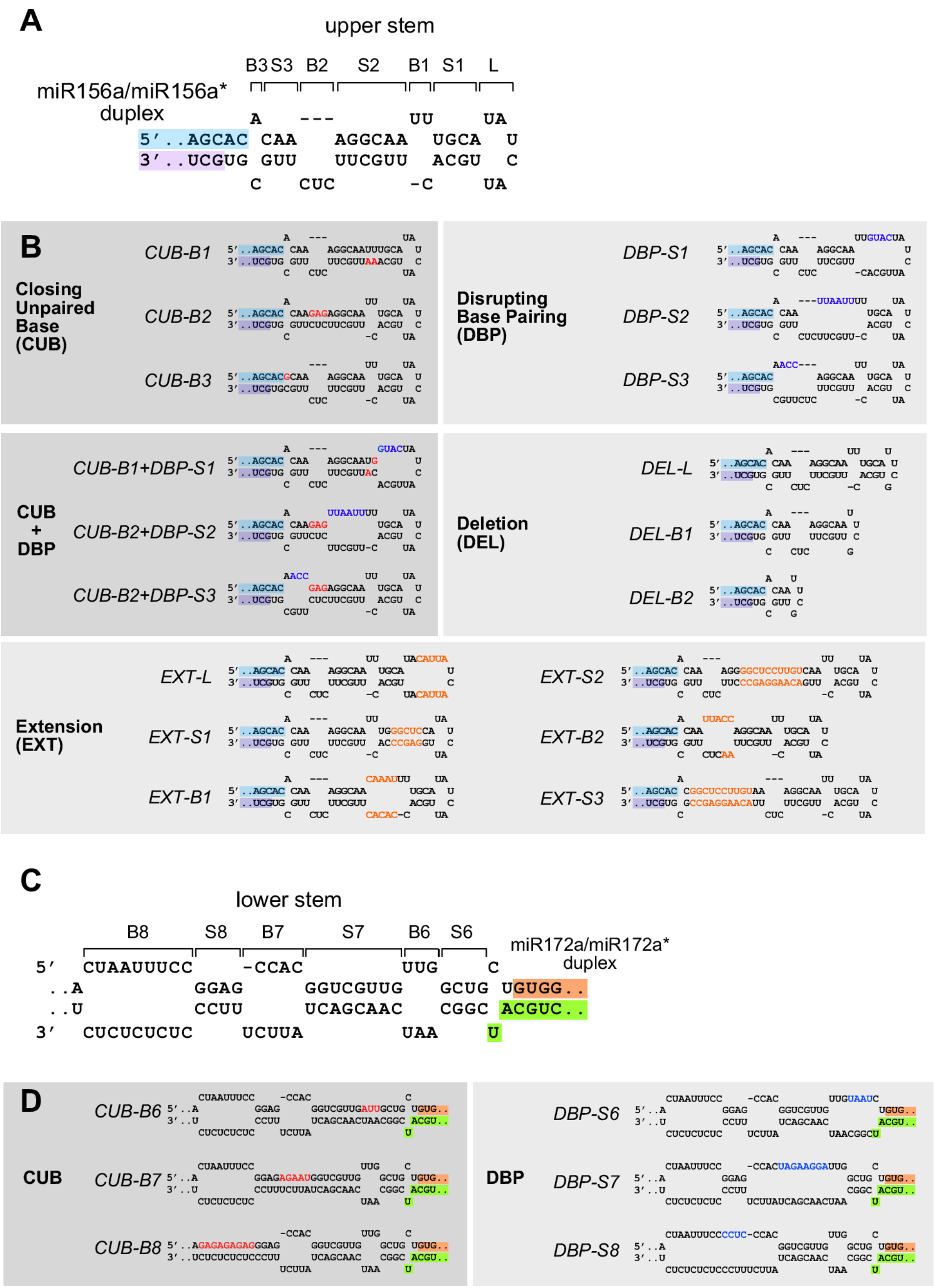

Supplementary Fig. S3. Schematic illustration of structural variants of pri-miR156a and pri-miR172a.

(A) Secondary structure of the pri-miR156a hairpin. The mature miR156a and miR156a* sequences are highlighted in cyan and purple, respectively. B, L, and S denote bulge, loop, and stem, respectively.

(B) A diagram of structural variants in the pri-miR156a upper stem. Partial miR156a/miR156a* sequences are shown. Mutated nucleotides are shown in different colors [closing unpaired bases (red), disrupting base-pairing (blue), and extension (orange)].

(C) Secondary structure of the pri-miR172a hairpin. Partial sequences of the miR172a/miR172a* duplex are highlighted in light green and orange, respectively. B, L, and S denote bulge, loop, and stem, respectively.

(D) Diagram of structural variants in the pri-miR172a lower stem. Partial sequences of the miR172a/miR172a* duplex are highlighted in light green and orange, respectively. Mutated nucleotides are shown in different colors [closing un-paired bases (red) and disrupting base-pairing (blue)].

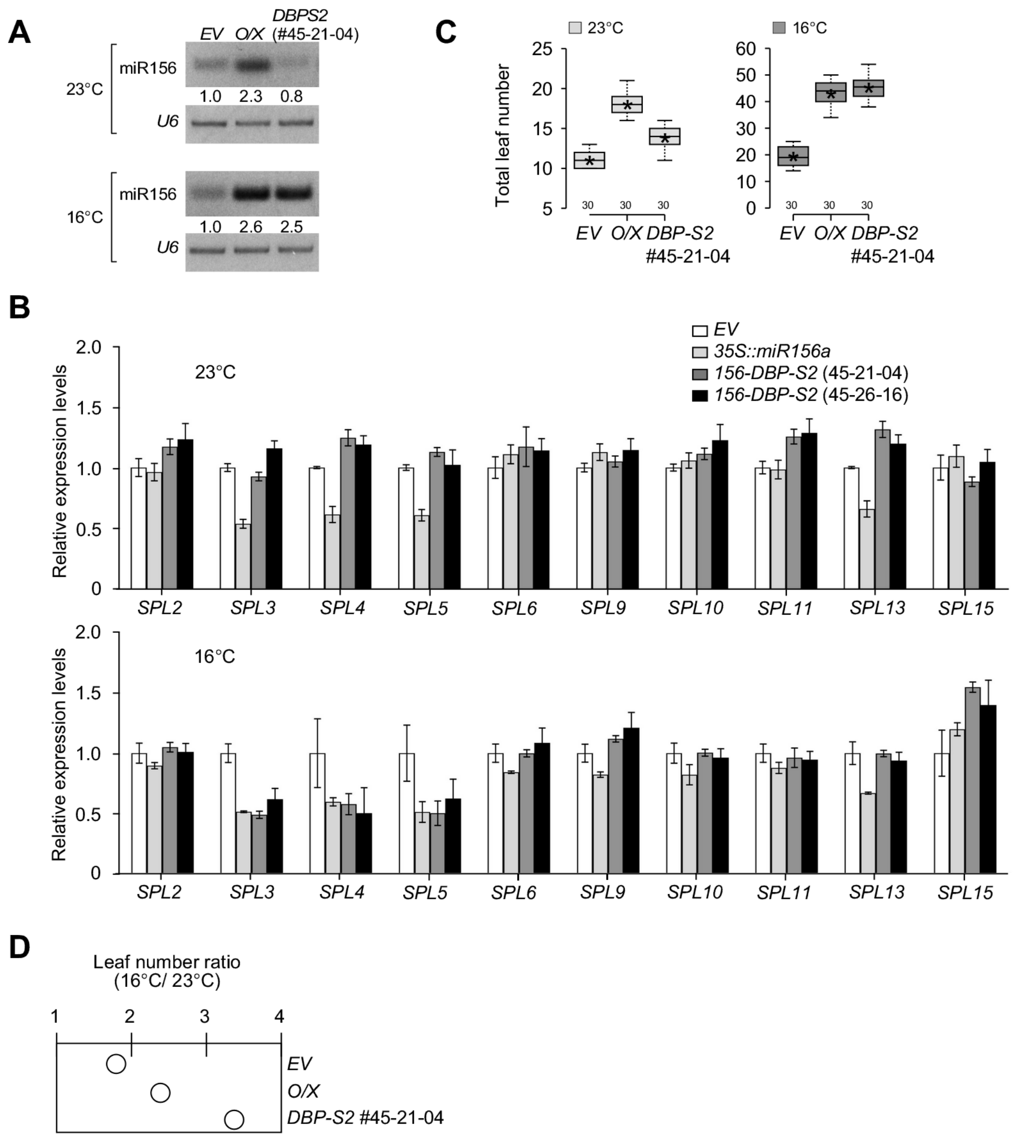

Supplementary Fig. S4. Analysis of an independent line (#45-21-04) of *156-DBP-S2* plants.

(A) Levels of mature miR156 in an independent homozygous line of *156-DBP-S2* (#45-21-04) plants at 23°C and 16°C. *U6* was used as a loading control. The numbers below each band indicate the fold change relative to the miR156 level in *EV* plants after normalization to *U6* at each temperature. (B) Expression levels of *SPL* genes in 9-day-old seedlings of #45-21-04 lines of *156-DBP-S7* plants at 23°C and 16°C, determined via qPCR. Note that the #45-26-16 line was the reference line used for *156-DBP-S2* plants in the main figures. Error bars indicate standard deviation. Each gene's expression levels in *EV* plants were set to one. (C) Leaf numbers of the #45-21-04 line of *156-DBP-S2* plants at 23°C and 16°C. Distribution of leaf numbers is shown as a box plot (see methods for further information for box

plots). *O/X*: plants overexpressing the un-mutated construct (*35S::miR156a*). (D) Leaf number ratio (16°C/23°C) of the #45-21-04 line of *156-DBP-S2* plants.

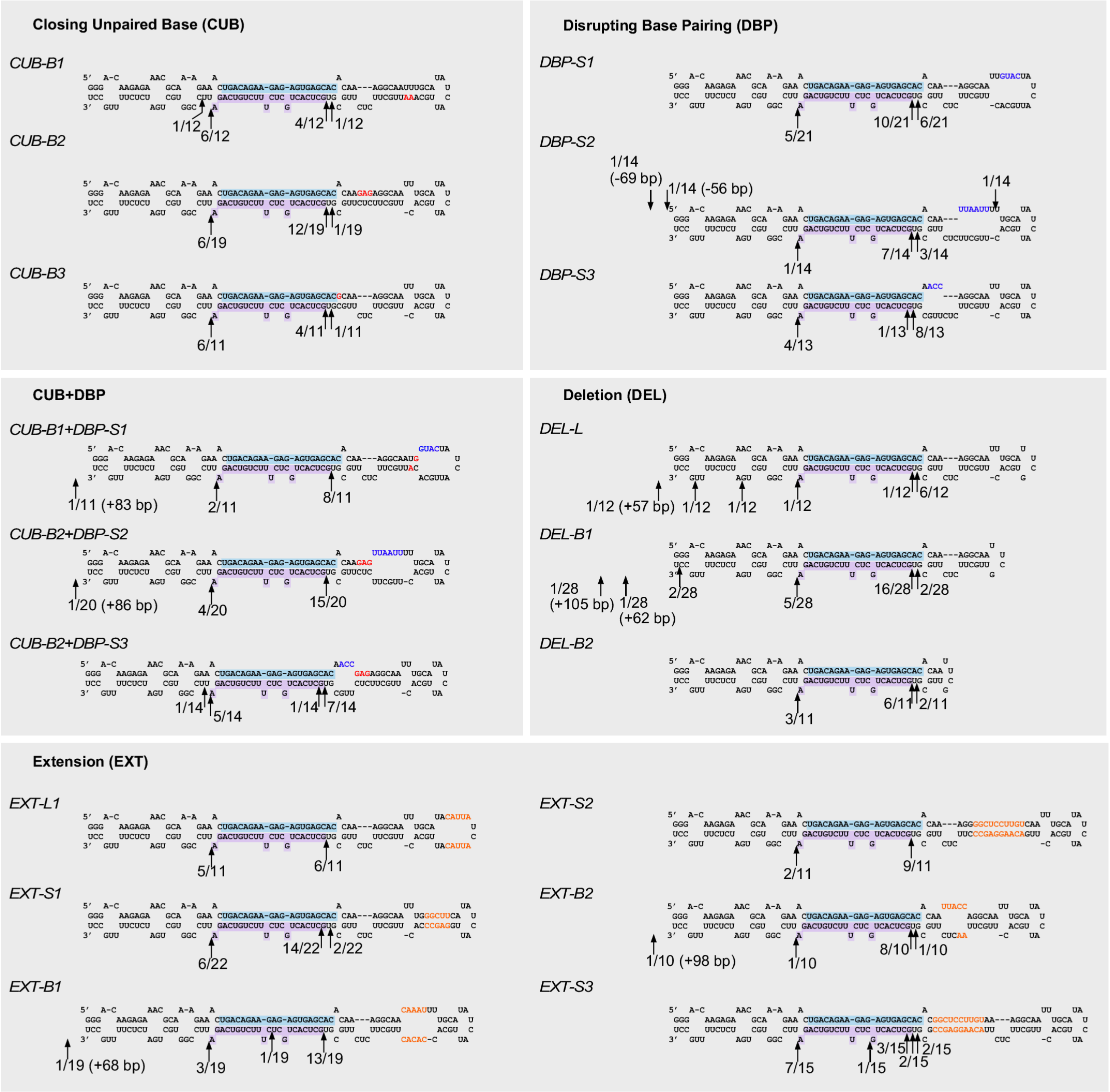

Supplementary Fig. S5. Map of cleavage sites of structural variants in the pri-miR156a upper stem detected by 5′-RLM-RACE. The mature miR156a and miR156* sequences are highlighted in cyan and purple, respectively. Mutated nucleotides are shown in different colors [closing unpaired bases (red), disrupting base-pairing (blue), and extension (orange)]. The arrows indicate the end of amplicons with the fraction of sequenced clones corresponding to each site. The numbers below arrows indicate the number of sequenced clones corresponding to each site.

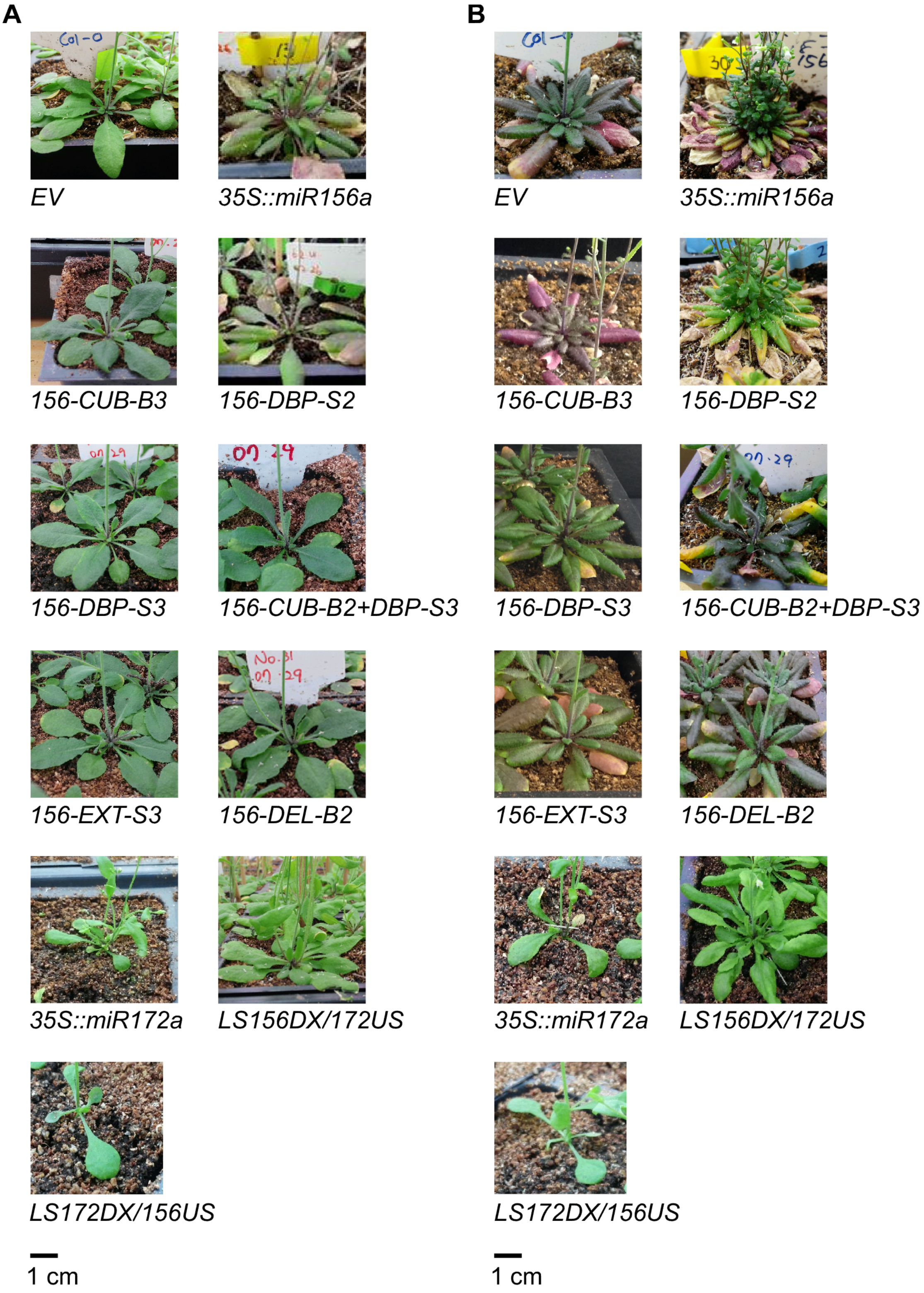

Supplementary Fig. S6. Flowering time phenotypes of pri-miR156a upper stem variants at 23°C (A) and at 16°C (B).

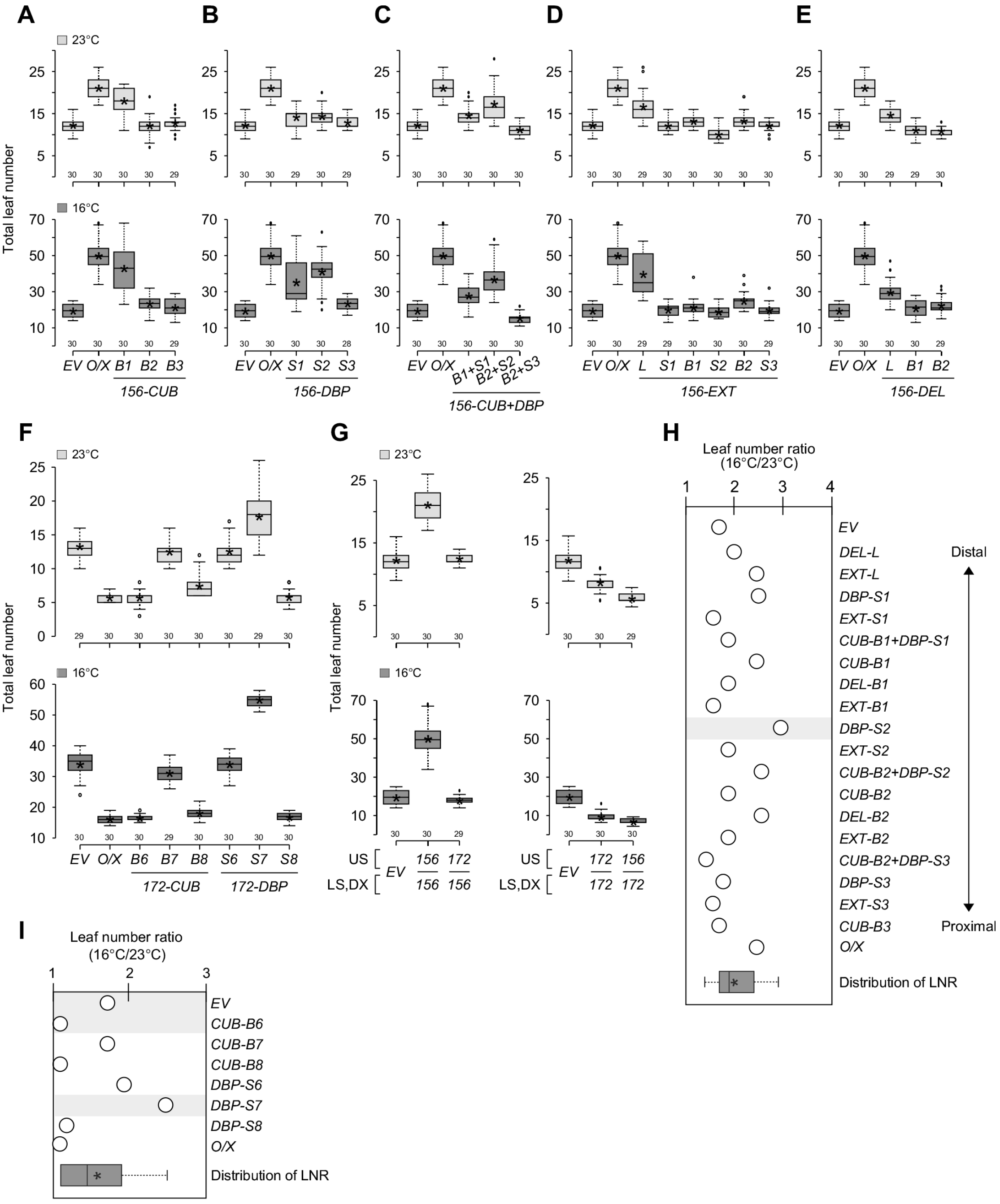

Supplementary Fig. S7. Flowering time of plants expressing structural variants of pri-miRNAs in the T_1 generation. (A-E) Leaf numbers of plants carrying structural variants in the pri-miR156a upper stem. *O/X*: plants overexpressing the un-mutated construct (*35S::miR156a*). (F) Leaf numbers of plants carrying structural variants in the pri-miR172a lower stem. *O/X*: plants overexpressing the un-mutated construct (*35S::miR172a)* (G) Leaf numbers of plants carrying stem-swapping variants of pri-miR156a and pri-miR172a at 23°C and 16°C. Distribution of leaf numbers is shown as a box plot (see methods for further information on box plots). (H) Leaf number

ratio (16°C/23°C) of plants overexpressing structural variants of pri-miR156a. (I) Leaf number ratio (16°C/23°C) of plants overexpressing structural variants of pri-miR172a. Distribution of LNR of the plants presented in this graph is shown as a box plot at the bottom of the graph.

Comparison	23°C	16°C
EV – *35S::miR156a*	1.0×10^{-32}	1.2×10^{-27}
35S::miR156a – *CUB-B1*	6.3×10^{-9}	0.23
CUB-B1 – *CUB-B2*	1.8×10^{-26}	1.1×10^{-31}
CUB-B2 – *CUB-B3*	2.4×10^{-11}	3.0×10^{-13}
35S::miR156a – *DBP-S1*	0.4	8.2×10^{-5}
DBP-S1 – *DBP-S2*	4.5×10^{-17}	8.1×10^{-5}
DBP-S2 – *DBP-S3*	1.6×10^{-4}	1.6×10^{-12}
35S::miR156a – *CUB-B1+DBP-S1*	7.7×10^{-18}	2.4×10^{-22}
CUB-B1+DBP-S1 – *CUB-B2+DBP-S2*	0.1	1.7×10^{-19}
CUB-B2+DBP-S2 – *CUB-B2+DBP-S3*	2.9×10^{-10}	3.6×10^{-24}
35S::miR156a – *EXT-L*	8.6×10^{-6}	9.3×10^{-8}
EXT-L – *EXT-S1*	6.9×10^{-11}	4.3×10^{-9}
EXT-S1 – *EXT-B1*	5.8×10^{-9}	0.04
EXT-B1 – *EXT-S2*	6.4×10^{-3}	1.2×10^{-3}
EXT-S2 – *EXT-B2*	1.6×10^{-4}	2.1×10^{-6}
EXT-B2 – *EXT-S3*	2.6×10^{-8}	0.1
35S::miR156a – *DEL-L*	3.9×10^{-27}	4.6×10^{-22}
DEL-L – *DEL-B1*	2.0×10^{-4}	4.8×10^{-5}
DEL-B1 – *DEL-B2*	0.78	1.3×10^{-8}

Comparison	23°C	16°C
EV – *35S::miR156a*	1.0×10^{-32}	1.2×10^{-27}
35S::miR156a – *LS156DX/172US*	5.0×10^{-33}	1.1×10^{-34}
EV – *35S::miR172a*	4.6×10^{-22}	2.2×10^{-20}
35S::miR172a – *LS172DX/156US*	0.07	0.6

Comparison	23°C	16°C
EV – *35S::miR172a*	3.1×10^{-27}	5.0×10^{-41}
35S::miR172a – *CUB-B6*	1.4×10^{-10}	2.1×10^{-9}
35S::miR172a – *CUB-B7*	2.6×10^{-18}	5.5×10^{-21}
35S::miR172a – *CUB-B8*	0.03	1.2×10^{-3}

Comparison	23°C	16°C
35S::miR172a – *DBP-S6*	4.9×10^{-10}	7.4×10^{-36}
35S::miR172a – *DBP-S7*	3.0×10^{-40}	5.4×10^{-44}
35S::miR172a – *DBP-S8*	2.1×10^{-21}	1.1×10^{-11}

Supplementary Fig. S8. Student`s *t*-test values between leaf numbers of mutant lines used in this study. Note that values smaller than 0.005 are shaded in grey.

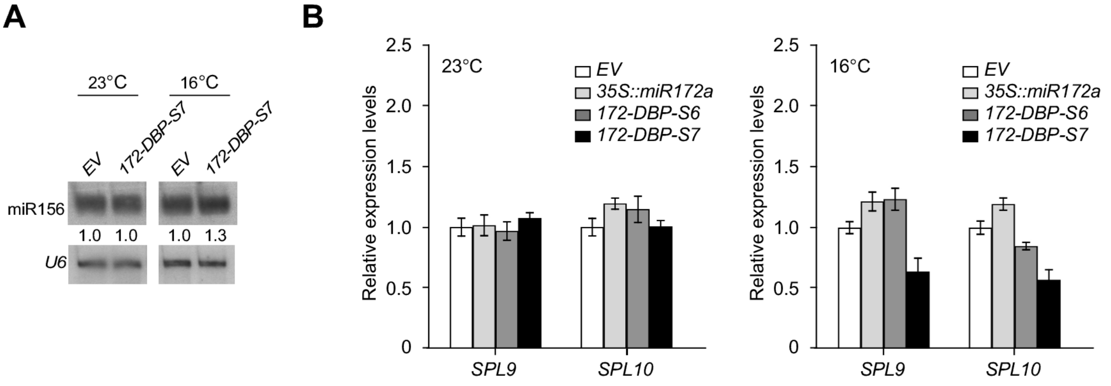

Supplementary Fig. S9. Analysis of *172-DBP-S7* plants.

(A) Levels of mature miR156 in *172-DBP-S7* plants at 23°C and 16°C. *U6* was used as a loading control. The numbers below each band indicate the fold change relative to the miR156 level in *EV* plants at 23°C after normalization to *U6* at each temperature. (B) Expression levels of *SPL9* and *SPL10* in *172-DBP-S6* and *172-DBP-S7* plants at 23°C and 16°C, determined via qPCR. Error bars indicate standard deviation. Expression levels of each gene in *EV* plants were set to one.

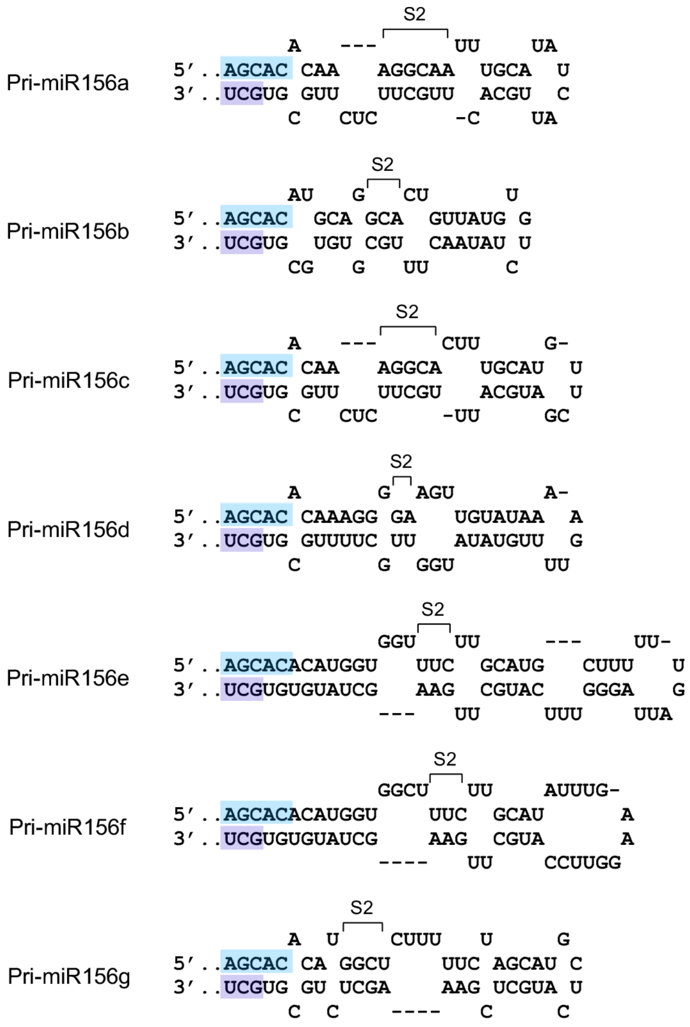

Supplementary Fig. S10. Comparison of sequences and structures of the upper stem region of different pri-miR156 genes.

Supplementary Table S1. Oligonucleotide sequences used in this study

Gene	Name	Sequence (5′ to 3′)	Purpose	Direction
Pri-miR156a	JH7066	GCTCTCACAATTTCCAAGGTGCTGC	5′-RACE for cleavage site mapping cleavage abundance	Anti-sense (outer)
	JH7067	AGGGCTCTTCCTTAAGGACAGTATTTGTG	5′-RACE for cleavage site mapping cleavage abundance	Anti-sense (inner)
	JH6193	cgattctagaGGGTCTCAAATGGAATCTCTTCTC	Absolute quantification (cloning pri-miR156a)	Sense
	JH6194	cgatggtaccGCCAGAGTTTAGATCGTATCTTC	Absolute quantification (cloning pri-miR156a)	Anti-sense
	JH5241	GATCTCTGAAGTTGGACTAATT	Absolute quantification (qPCR)	
	JH5242	AGACAGAGAAAGATTGTGTAAG	Absolute quantification (qPCR)	Anti-sense
Pri-miR156b	JH6195	cgattctagaTCGACTGTTTCGCCATTAGA	Absolute quantification (cloning pri-miR156b)	Sense
	JH6196	cgatggtaccGTAAGCAAGCACCCACTTCC	Absolute quantification (cloning pri-miR156b)	Anti-sense
	JH5244	TCCATCTAGGTTTTTTTGAATTAGT	Absolute quantification (qPCR)	Sense
	JH5245	CGATCAAAGAAAGAAATGTCTAA	Absolute quantification (qPCR)	Anti-sense
Pri-miR156c	JH6197	cgattctagaACTTTCCTCTTCTCCTTCGGTTA	Absolute quantification (cloning pri-miR156c)	Sense
	JH6198	cgatggtaccAAATCAATGGCAGCAAATAAGAA	Absolute quantification (cloning pri-miR156c)	Anti-sense
	JH5248	CAGAATTTGTGTCGGTAAGG	Absolute quantification (qPCR)	Sense
	JH5249	GATCGTATTTAATTTACCTTTGAG	Absolute quantification (qPCR)	Anti-sense
Pri-miR156d	JH7082	ACCCTAACTTGCAGTGTCCGTA	Absolute quantification (cloning pri-miR156d)	Sense
	JH5253	CATAACTAGAACAATGGAATAAGG	Absolute quantification (cloning pri-miR156d)	Anti-sense

			Absolute quantification (qPCR)	
	JH5252	TTTAGTGTCATAACTAGATAAATATATG	Absolute quantification (qPCR)	Sense
SPL2	JH6034Q	ACGGGTTGGAGGTTGCTTGAGG	qPCR	Sense
	JH6035Q	TTTCCGATACCGAGCACAATAG	qPCR	Anti-sense
SPL3	JH6036Q	CTTAGCTGGACACAACGAGAGAAGGC	qPCR	Sense
	JH6037Q	GAGAAACAGACAGAGACACAGAGGA	qPCR	Anti-sense
SPL4	JH6038Q	TCAAGGGTAGAGATGACACTTCCTATGC	qPCR	Sense
	JH5652	GGTTCAAGTATAGTCTTAGCGTTTGCAT	qPCR	Anti-sense
SPL5	JH6040Q	CCAGACTCAAGAAAGAAACAGGGTAGACAG	qPCR	Sense
	JH6041Q	TCCGTGTAGGATTTAATACCATGACC	qPCR	Anti-sense
SPL6	JH6042Q	CCACCGTACAAGTAGACTCGTGAG	qPCR	Sense
	JH6043Q	GAGATTTTGGTTGGGTTGGGTGA	qPCR	Anti-sense
SPL9	JH6130Q	TTTAGTCAAGGTTCAGTTGGTGGA	qPCR	Sense
	JH6131Q	CGCCATGTATTGTTGTTGTTGTT	qPCR	Anti-sense
SPL10	JH6046Q	GTGTGGGAGAATGCTCAGGAGG	qPCR	Sense
	JH6047Q	ACGGGAGTGTGTTTGATCCCTTGTG	qPCR	Anti-sense
SPL11	JH6048Q	AGTCCAAGTTTCAACTTCATGGCG	qPCR	Sense
	JH6049Q	GAACAGAGTAGAGAAAATGGCTGCAC	qPCR	Anti-sense
SPL13	JH6050Q	CCAATCTCTTCTTCTCCAAACAGTACCAGAAGC	qPCR	Sense
	JH6051Q	GAAGCAAATGAGGGACTGACGACG	qPCR	Anti-sense
SPL15	JH6052Q	GAATGTTTTATCACATGGAAGCTC	qPCR	Sense
	JH6053Q	TCATCGAGTCGAAACCAGAAGATG	qPCR	Anti-sense
AP2	JH4587	ACAACAAGATTCTCTCCACTCTAATGA	qPCR	Sense
	JH4588	GCAGCCAATTTTGATGAGGAGTA	qPCR	Anti-sense
SMZ	JH6225	CATCATCATCGGAAAGTATAAAGTTGAC	qPCR	Sense
	JH6226	GTCTTCAGAGGTTTCATGGTTGCCATG	qPCR	Anti-sense
SNZ	JH6173	CAGCAGCAGCAAAATGCAATGAG	qPCR	Sense
	JH6174	CACCGATCGATTCAAACCCATGT	qPCR	Anti-sense
TOE1	JH4583	TTTACTGGAACGGAGCATGC	qPCR	Sense
	JH4584	GTGTGGATAAAAGTAACCACGTGTT	qPCR	Anti-sense
TOE2	JH4589	GGCATGTGATACGCCTTTCA	qPCR	Sense
	JH4590	ATAGAGACCGGGCTGATTCAGAT	qPCR	Anti-sense
TOE3	JH4591	TATGATAAAGCGGCAATAAAGTGT	qPCR	Sense
	JH4592	AGGAATGCGGTAAGGGGAAG	qPCR	Anti-sense
AT1G13320	JH6505	GCGGTTGTGGAGAACATGATACG	qPCR (reference gene)	Sense
	JH6506	GAACCAAACACAATTCGTTGCTG	qPCR (reference gene)	Anti-sense
AT2G28390	JH7588	TTGATCCACTTGCAGACAAGGC	qPCR (reference gene)	Sense
	JH7589	TACCCTTTGGCACACCTGATTG	qPCR (reference	Anti-sense

	gene)

*Lowercase letters denote synthetic restriction enzyme sites.

References

Ramakers C, Ruijter JM, Deprez RH, Moorman AF. 2003. Assumption-free analysis of quantitative real-time polymerase chain reaction (PCR) data. *Neurosci Lett* **339**, 62-66.
